# Tissue-based diagnosis of systemic amyloidosis: Experience of the informal diagnostic center at Uppsala University Hospital

**DOI:** 10.48101/ujms.v127.8913

**Published:** 2022-09-27

**Authors:** Justina Damjanovic Vesterlund, Elisabet Ihse, Ulrika Thelander, Alice Zancanaro, Gunilla T. Westermark, Per Westermark

**Affiliations:** a Clinical Pathology, Uppsala University Hospital; b Department of Genetics, Immunology and Pathology; c Department of Medical Cell Biology, Uppsala University, Uppsala, Sweden

**Keywords:** Amyloidosis, systemic, monoclonal antibodies, subcutaneous fat tissue, biopsy

## Abstract

Diagnosis of systemic amyloidosis is a clinical challenge and usually relies on a tissue biopsy. We have developed diagnostic methods based on the presence of amyloid deposits in abdominal subcutaneous fat tissue. This tissue is also used to determine the biochemical type of amyloidosis, performed by western blot and immunohistochemical analyses with the aid of in-house developed rabbit antisera and mouse monoclonal antibodies. Mass spectrometric methods are under development for selected cases. The diagnostic outcome for 2018-2020 was studied. During this period, we obtained 1,562 biopsies, of which 1,397 were unfixed subcutaneous fat tissue with varying degrees of suspicion of systemic amyloidosis. Of these, 440 contained amyloid deposits. The biochemical nature of the amyloid was determined by western blot analysis in 319 specimens and by immunohistochemistry in further 51 cases.

## Introduction

Systemic amyloidoses are generally life-threatening diseases, earlier believed to be very rare but are now increasingly often diagnosed. Several therapeutic drugs have been developed during the last years, and the interest in the disorders is steadily growing.

Amyloid is characterized by misfolding of proteins that aggregate into cross β-sheet fibrils (1). In humans, such fibrillar deposits are mainly extracellular, exerting detrimental effects on cells, which may undergo apoptosis or necrosis. Close to 40 different human proteins have been identified as amyloid forming in vivo, and more are expected to be discovered (2). The proteins are usually small (below 200-300 amino acid residues long) and vary substantially in their native folding. Despite this, irrespective of biochemical origin, the fibrils are very similar in molecular arrangement, appearance, and properties.

Amyloid can be localized or systemic. The amyloidogenic protein in the systemic forms, where deposits appear in many different organs, is synthesized by one or a few cell types and transported in a native or near-native form via the bloodstream to a target place, where the typical fibrils form. There is an increasing understanding of how a single protein develops into a fibril. A missense mutation is a common cause of misfolding, characteristic of the many hereditary types of amyloidosis. However, a mutation is not a prerequisite, and wild-type proteins can also be amyloidogenic. The spreading of amyloid likely depends on seeding, by which a misfolded protein or protein aggregate recruits molecules of the same kind, catalyzes their misfolding, and propagates the formation of fibrils (3).

Presently, 17 different proteins are known to give rise to systemic amyloidoses (2). Three biochemically different systemic amyloidoses are predominating. AL amyloidosis, where the fibril protein is derived from a monoclonal immunoglobulin light chain, depends on a plasma cell clone, often small and usually in the bone marrow. The fibril protein in AA amyloidosis is a large N-terminal fragment of the acute phase protein serum amyloid A (SAA), expressed by the liver at acute or chronic inflammations. The third of the most prevalent systemic amyloidoses is of a transthyretin (TTR) nature. Plasma TTR, mainly synthesized by the liver, forms the fibrils in most hereditary forms of amyloidosis, but wild-type TTR is also amyloidogenic and is the fibril protein in the age-associated wild-type (ATTRwt) amyloidosis, earlier called senile systemic amyloidosis (4).

Symptoms from systemic amyloidoses vary considerably in all biochemical forms. In many types of the disease, polyneuropathy, cardiomyopathy, cardiac arrhythmia, or renal problems are common, but virtually all organs may be affected in various combinations. This variability, in combination with the relative rarity of systemic amyloidosis, makes the diagnosis difficult (5), and many patients meet several doctors before a diagnosis can be established (6). Coming to a definite diagnosis can take considerable time, which is unfortunate since late diagnosis is associated with less successful treatment results. The problem is particularly severe in AL amyloidosis.

For more than 20 years, we have built up a diagnostic facility center at Uppsala University Hospital. The present paper describes the procedures and their background used at our laboratory in Uppsala. We report on some trends seen by our biopsy material over the years and analyze the results more in detail for the latest 3-year period for which all data are available. The study was conducted in agreement with the Declaration of Helsinki and does not need an ethical approval according to the Swedish Ethics Review Authority (Dnr. 2021-06773-01).

## Tissue-based diagnosis of amyloidosis in Uppsala

### Development of subcutaneous abdominal fat biopsy for diagnosis of amyloidosis

When comprehensive studies on systemic amyloidosis started, the AA variant dominated in Sweden and other European countries, and this disease most often followed rheumatic diseases. The interest in amyloidosis was low, and AA amyloidosis was often missed clinically, even at autopsy (7). A finding that AA amyloid deposits usually occur around fat cells in the subcutaneous tissue (8), initially described in 1909 (9) but later denied (10), prompted us to study whether fine-needle aspiration biopsy could be a method to obtain a correct diagnosis of amyloidosis. Since the result was successful in the single studied patient (11), we extended the study to more patients and showed that the method is a risk-free, reliable, and straightforward alternative to rectal biopsy, usually used at that time (12, 13). Fine-needle biopsy of abdominal fat tissue later became the most common initial method to diagnose systemic amyloidosis worldwide (14-16).

**Figure 1:**
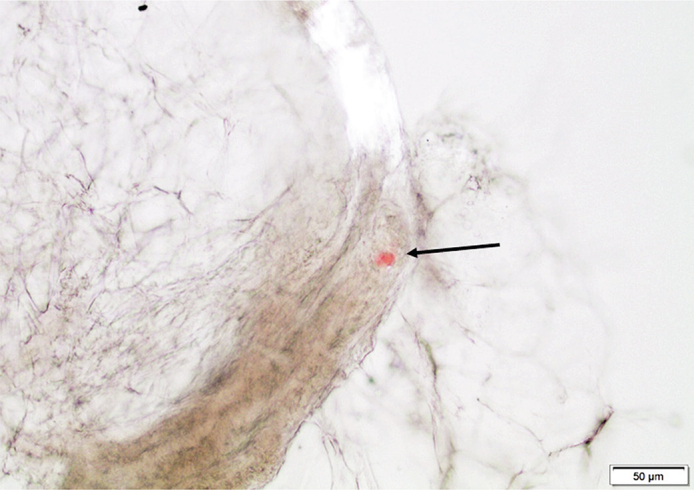
Abdominal fat tissue fragment in a squeeze preparation containing a very small ATTR particle (arrow), stained with Congo red. Bar = 50 μm.

While fine needle aspiration biopsy of subcutaneous fat tissue is an excellent method to diagnose AA and AL amyloidosis, it is less successful for the ATTR forms, particularly wild-type (wt). One reason is that the amount of ATTR amyloid in fat tissue is often sparse. However, an even more important explanation for the results with fine needle aspiration is that the ATTR deposits are associated with collagen that is not easily included in the aspirated material. For this reason, we have largely discontinued the use of fine needle aspiration biopsy from subcutaneous tissue for amyloidosis diagnosis. We now recommend a slightly more invasive method for this tissue. We first introduced a small surgical biopsy (about 1 cm^3^) (17). Further development has been the introduction of punch biopsies, including subcutaneous tissue (18). Demonstration of small amyloid deposits in sections of fat tissue is often difficult. We, therefore, introduced squeeze preparations of unfixed tissue, which has made the identification of deposits much more successful (19, 20). One further advantage of this method is that some types of amyloid, e.g. ATTRwt, stain better with Congo red when not going through formalin fixation and paraffin embedding steps. Minimal amyloid deposits can be identified in squeeze preparations (Figure 1).

### Determination of amyloid type

The specific treatments for amyloidosis require the determination of the exact type of disease. Consequently, there is a big need for safe methods to diagnose amyloidosis and determine its type. These demands have resulted in the more or less independent development of different methods at several specialized laboratories in the world (21). Variants of immunohistochemistry (IHC) and, later, mass spectrometry (MS) are most commonly used.

Fine needle biopsy of abdominal fat tissue can be used for the determination of the biochemical type of amyloidosis. The first example of this was probably performed in Uppsala in 1979, when double immunodiffusion was utilized to identify protein AA in micro-extracts of subcutaneous adipose tissue (22). Some laboratories still use subcutaneous fat tissue obtained by fine needle aspiration for typing, but now with a modern, mass spectrometric technique (23, 24).

### Immunological methods used in Uppsala

#### Development of specific antibodies

It is a general experience that most commercial antibodies are unsuitable for clinical diagnostic work of amyloidosis (21, 25, 26). One reason for this failure is that such antibodies usually are raised against normally folded proteins. Therefore, it is necessary to develop antibodies that react not only with natively folded proteins but also with their amyloid fibril form. For polyclonal antibodies, this can be done by using partly degraded and thereby solubilized fibrils as immunogens (27, 28) or by raising antibodies against short peptides corresponding to a part of the protein incorporated in the amyloid (29). Recombinant protein may also be used. The antibodies we developed for amyloid typing have been raised against various antigens (Table 1). Initially, we developed rabbit antisera, of which we are still using some but we are trying to replace these gradually with mouse monoclonal antibodies (mabs), the first being anti-protein AA mab Sne5 from 1995 (unpublished) and the latest anti-AL kappa mab pwkap (to be published). The most important reason to leave polyclonal antibodies is the indefinite availability and the uniform properties of mabs.

**Table 1:** Rabbit antisera and mouse monoclonal antibodies developed for clinical typing of human systemic amyloidoses

Antibody	Fibril protein	Immunogene	Antibody type	Antibody used in	Reference
Sne5	AA	Purified protein AA	m-mab	IHC	Unpublished
A126	AA	Peptide	pc, rabbit	wb	(17)
A147	AL, κ	Peptide	pc, rabbit	wb	(17)
pwkap	AL, κ	DAM	m-mab	IHC, wb	Unpublished
pwlam	AL, λ	DAM	m-mab	IHC, wb	(55)
1898	ATTR	Recombinant TTR50-127	pc, rabbit	IHC, wb	(56)
7X	ATTR	DAM	m-mab	IHC, wb	(57)
A82/01	AApoAIV	Peptide	pc, rabbit	IHC	(56)
A159	AApoAI	Peptide	Pc, rabbit	IHC	(58)

DAM: alkali-degraded amyloid; Pc: polyclonal; m-mab: mouse monoclonal antibody; IHC: immunohistochemistry; wb: western blot.

#### Western blot analysis

As described above, we obtain unfixed fat tissue specimens, and this has a historical explanation. Our earliest trials to establish the type of systemic amyloidosis were by double immunodiffusion or by amino acid sequence analysis (30). Later, after attempts with enzyme-linked immunosorbent assay (ELISA) (17), we adopted sodium dodecylsulfate-polyacrylamide gel electrophoresis (SDS-PAGE) with western blot analyses of extracts of subcutaneous fat tissue (19, 20), which we found to be a more sensitive method. This is still the method that we use for all adipose samples, except for those with very small amyloid deposits. For routine clinical analyses, we use the antisera 1898, A126, A147, and the mabs pwlam and pwkap (Table 1), always with known control materials that often give characteristic band patterns. The antisera 1898 and A126 react with all ATTR and AA materials, respectively, if there is enough amyloid material in the fat tissue (≥score 1-1.5 on a scale of 0-4). A147, directed against a linear epitope at the beginning of the constant region of AL kappa, labels at least most cases of this type. The mab pwlam reacts with a not fully identified epitope at the junction between the variable and constant region in AL lambda proteins. This antibody has been validated by IHC, showing 50% sensitivity and 100% specificity for AL lambda amyloid (31). The sensitivity by western blot analysis is higher in our experience, although no exact figure has been determined. In addition to these antibodies, we have generated other rabbit antisera, not reported here, which we use for unreactive materials.

#### Immunohistochemistry (IHC)

IHC has been and still is the most common method to determine the type of amyloidosis in most laboratories. IHC is used both on sections of formalin-fixed and paraffin-embedded materials and of frozen sections, the latter particularly in renal pathology.

We also use IHC for adipose tissue samples, particularly for ATTRwt materials. The subcutaneous amount of amyloid is often very low in this amyloid type, and it is common to find only one or very few but usually distinctive deposits in the squeeze preparations. Western blot or mass spectrometry are not helpful for such biopsies. Removing the cover glass followed by IHC with our monoclonal ATTR antibody 7X often solves the problem.

Like other clinical pathology departments working with amyloidosis, we receive paraffin blocks of tissue with identified amyloid deposits to determine the biochemical type. Usually, there is a substantial amount of amyloid in such specimens. We use traditional IHC but with our antibodies. AA and ATTR cases offer no problems. Approximately 75% of AL cases can be diagnosed, and with the recent development of an AL kappa mab pwkap, we expect the number of undiagnosed cases to decrease.

### Mass spectrometry

Mass spectrometry for the determination of amyloid type was pioneered by researchers at the Mayo Clinic and is now an established method in many laboratories (32, 33). The technique is most commonly based on formalin-fixed and paraffin-embedded materials, from which amyloid is dissected with laser dissection microscopy (LDM). After solubilization, the crude material is enzymatically fragmented into peptides, usually with trypsin which cleaves after the basic amino acid residues arginine and lysine. The obtained MS data are after that analyzed by proteomics.

MS can also be used for non-fixed amyloid samples. Particularly, fat tissue obtained by fine needle aspiration is used for amyloid type determination in some laboratories, as mentioned above.

Most laboratories using IHC for type determination turn to MS as a next option when IHC results are uncertain. Since we receive surgically or punch adipose tissue biopsies, we have the opportunity to directly extract these for MS. We are presently developing a method with initial SDS-PAGE followed by the analysis of low molecular protein bands in order to obtain a concentration of pertinent proteins.

## Trends in Uppsala biopsy materials 2006-2021

**Figure 2:**
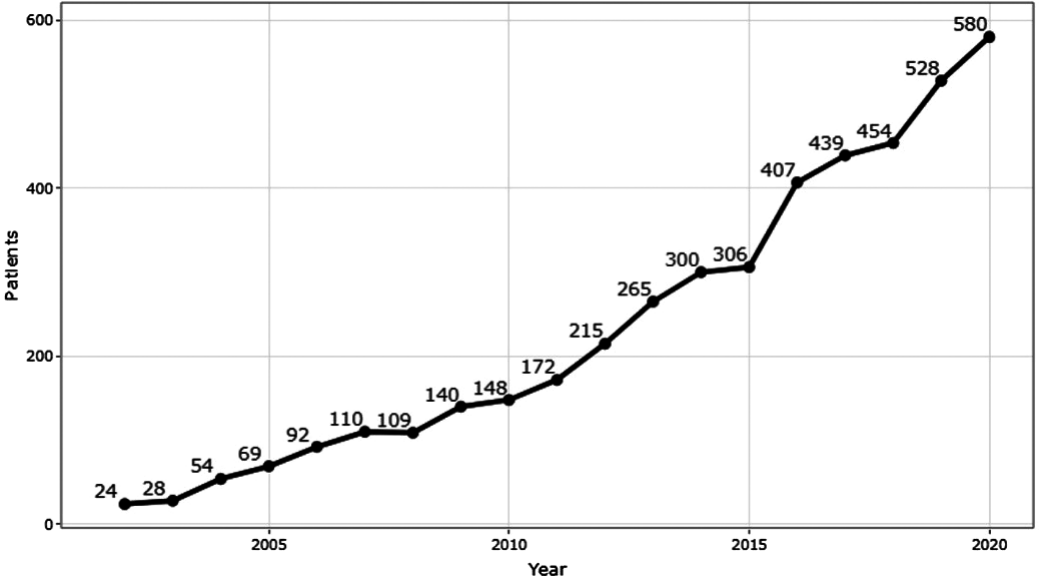
The number of biopsies obtained for amyloid diagnosis at the Uppsala laboratory for 2002-2020. There was a relatively even increase until 2015, with a more substantial increase during the last years.

Since our organized amyloid clinical work started, the number of obtained biopsies has increased steadily (Figure 2). Fresh fat tissue biopsies constitute the majority of them. For the three years 2018-2020, 1,397 samples (89.4%) were from subcutaneous fat tissue, obtained either surgically or with a punch. With a few exceptions, the biopsies were taken without a previous proven diagnosis of amyloidosis. The remaining 165 biopsies contained formalin-fixed and paraffin-embedded materials, diagnosed with amyloidosis elsewhere and sent to us to determine the type. These materials varied in nature and included endomyocardial, renal, liver, skin, and many other kinds of specimens.

### Subcutaneous fat tissue biopsies

**Figure 3:**
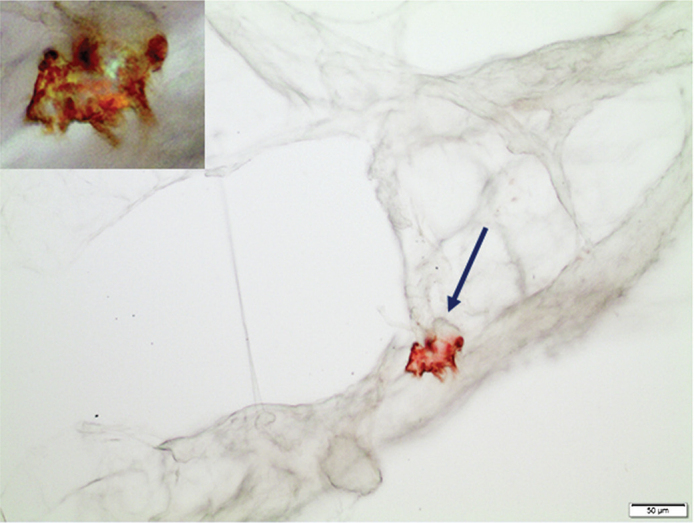
A sharply delined amyloid particle in a subcutaneous fat tissue biopsy. The amount of amyloid (arrow) was too sparse to analyze by western blot. Therefore, the Congo red stained squeeze preparation was subjected to IHC with our anti-ATTR antibody 7X, resulting in evident labeling of the deposit. Insert: the same particle at higher magnification, visualized in polarized light. Green-red-yellow birefringence due to the remaining Congo red staining is obvious. Immunolabeling was shown with 3,3’-diaminobenzidine. Bar = 50μm.

We have developed a unique principle way to diagnose systemic amyloidosis based on fat tissue biopsies, and the results for the three-year period (2018-2020) are shown in Table 2. About one-third of them (440) contained amyloid. The amount of amyloid varied, ranging from a situation with almost no normal tissue structures remaining to very tiny deposits. Western blot analysis revealed the fibril protein in 72.5% of the cases. Nineteen further biopsies were rich in amyloid but not possible to type with our antibodies. The remaining 102 biopsies contained small amounts of amyloid, often only as one or a few but distinct deposits. For further characterization of these materials, we applied IHC and used the original squeeze-prepared slides. Since the deposits often were minimal and could be difficult to find again (Figure 1), their coordinates in the microscope were noted before removing the cover glass. The slide was then taken back to water before IHC was performed in the usual way. In this way, it was possible to identify ATTR as the amyloid fibril protein in 51 cases (Figure 3). In 51 other cases which were highly suspected to be of ATTR nature, this method did not give a convincing result due to the minimal amount of amyloid, often only one dot-like deposit, a common finding in ATTR amyloidosis.

**Table 2:** Tissue biopsies for amyloid diagnosis 2018-2020

Total number of biopsies	1,562
Abdominal fat biopsies (unfixed)	1,397
Abdominal fat biopsies with amyloid	440 (31.5%)
Type determined (western)	319 (72.5%)
Type determined (IHC)	51 (11.6%)
Total number typed	370 (84.1%)
Type not determined	70 (15.9%)
Too little material	51*
Not classified amyloid type	19

* Almost all had microscopic appearance of ATTR amyloid and a disease history supporting ATTRwt.

**Figure 4:**
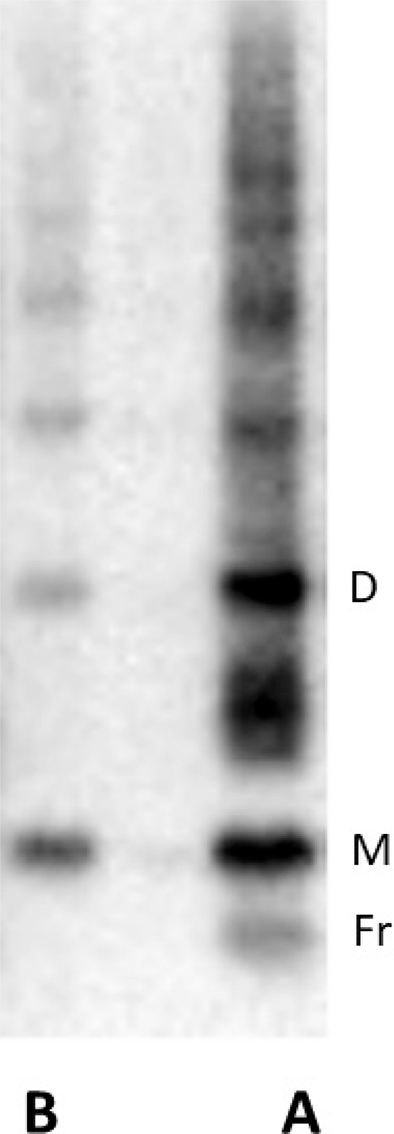
Typical findings at western blot analysis of ATTR amyloid containing fat tissue biopsies demonstrating TTR monomers (M) and dimers (D) as well as TTR fragments (Fr). Type A, with a mixture of full-length and fragments of TTR, seen to the right, is found in most mutated forms as well as in wild-type ATTR amyloidosis. Type B to the left is found in many patients with ATTRV30M amyloidosis.

**Type A and type B ATTR amyloidosis.** The ATTR antiserum 1898 was raised against recombinant TTR50-127. In contrast to a commercially available product, this antiserum reacts with both full-length and fragmented ATTR species (34, 35). By western blot analysis, we are able method, we are able to distinguish between ATTRv type A and type B fibrils (Figure 4). Patients with these variants behave like having two different disorders (36). Most importantly, patients with type A disease incorporate more wt TTR in their amyloid fibrils, often leading to progressive amyloid cardiomyopathy (37, 38), which is associated with shorter survival than patients with type B fibrils (36).

The appearance of the two patterns is particularly seen in patients with the *TTRV30M* mutations and is very rare in other variants. However, pattern A is by far the most common in other mutations than TTRV30M (39) and is the only one seen in ATTRwt amyloidosis (unpublished result).

We have tried to determine the ATTR fibril type in all fat pad biopsies with at least a moderate (1-1.5 + on a 0-4+ scale) amount of amyloid and were able to do so in 146 biopsies. While there were no patients with type A fibrils below the age of 50, the age spectrum among the type B patients was remarkably wide (mean age 53.7, range 28-88), with nine patients over 70 years and one as old as 88 years.

**Proportions of the three major systemic amyloidoses.** AL amyloidosis was the most prevalent type in our material, as in reports of most other amyloid laboratories. Notably, the amount of amyloid in AL materials was always moderate (2+) to very rich (4+). The rarity of AA amyloidosis with only 4 biopsies from patients with this type is in accordance with the findings in other western countries (40).

## Additional biopsy materials

In addition to the biopsies from subcutaneous adipose tissue, we received 165 materials, most formalin-fixed and paraffin-embedded, where amyloid had been found at other laboratories and sent to us for typing by IHC. These are not reported in detail here, but one notable aspect has come up repeatedly: a question regarding localized vs. systemic amyloidosis.

In almost all cases, fat tissue biopsies were taken due to suspicion of systemic amyloidosis. However, the question of a localized form arose in some situations. Most biopsies from localized amyloidoses were received as formalin-fixed and paraffin-embedded tissue blocks.

**Insulin amyloid**. Five cases of iatrogenic localized, insulin-derived (AIns) amyloid were identified in our 3-year material. There was no suspicion of this amyloid type in any of the patients, and four materials were obtained paraffin-embedded after that amyloid unexpectedly had been discovered in other pathology departments, while the fifth subcutaneous material was received unfixed and contained large amounts of amyloid.

Amyloid formation is not a rare effect of repeated subcutaneous injections of insulin at one site. For unknown reasons, insulin is then misfolded and deposited as fibrillary aggregates instead of being released into the circulation (41, 42). The deposits create one or several nodules containing heavy amyloid deposits and are strictly local except for one published case, in whom AIns amyloid was identified in draining lymph nodes (43). If the phenomenon is not known to the clinician or pathologist, the deposits may be misdiagnosed as a sign of systemic amyloidosis. Analysis of the amyloid with immunological or mass spectrometric methods elucidates the nature of the material. The formation of insulin-derived amyloid is likely an underappreciated event and may cause blood sugar control problems (44).

**Figure 5:**
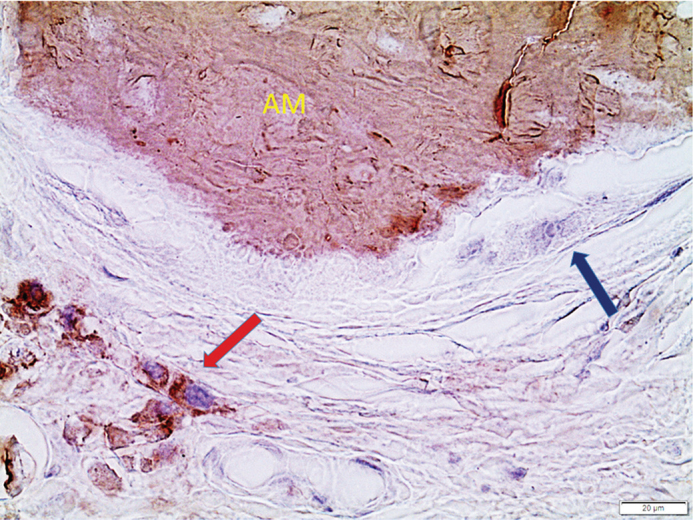
Localized AL amyloidosis of immunoglobulin light chain lambda origin. A sharply delimited immunolabeled amyloid particle (AM) is surrounded by multinuclear giant cells (black arrow) that are often difficult to recognize. Groups of immunolabeled lambda-light chain plasma cells (red arrow) are evident. These characteristics make it possible to suggest that the process is localized and not part of systemic disease. Anti-AL antibody pwlam binding visualized with 3,3’-diaminobenzidine. Bar = 20 μm.

**AL amyloidosis** occurs both as localized and systemic disorders, and the distinction is essential since while the systemic form is life-threatening, the localized is usually not. Localized AL amyloidosis develops at a site of clonal expansion of plasma cells secreting an amyloidogenic immunoglobulin light chain that forms fibrils close to the production site. Localized AL amyloidosis rarely develops into a systemic disease (45). Localized AL amyloid does often, but not always, have a characteristic microscopic appearance with bright birefringent deposits, the presence of groups of plasma cells, and giant cells that may cover a surface of single amyloid particles (Figure 5) (46). Even at light microscopy, the deposits can look fibrillary and organized. A number of localized AL amyloidosis specimens are sent to us each year from other pathology laboratories for type-determination.

## Discussion

Systemic amyloidoses are all comparably rare and often diagnosed several years after the onset of the disease. A correct diagnosis, including the amyloid type, is required for proper treatment. Treatment options for the different variants have emerged rapidly during the last years (47, 48), and the demands for an accurate diagnosis have significantly increased. The clinical diagnosis of amyloidosis is still based on tissue biopsy. Amyloid is often easy to recognize microscopically, but there are many pitfalls. The usual way is to stain tissue material with the dye Congo red, introduced in amyloid diagnostics one hundred years ago, nowadays in combination with polarization microscopy. Although principally simple, the staining procedure has to be strictly controlled since other tissue components can look like amyloid in overstained materials. Experience is necessary, and the concentration of amyloid diagnostics to a limited number of examiners is, therefore, important. This is even more apparent regarding the type-determination of amyloid deposits. To overcome the difficulties in diagnosing systemic amyloidosis and develop the most efficient treatment procedures for the different variants, referral centers for systemic amyloidosis have been established in many countries, particularly in Europe, USA, Japan, and Australia. In Sweden, a center for caring for patients with ATTRv amyloidosis was set up in Umeå decades ago. Patients with all other types of systemic amyloidosis have been investigated and treated at local hospitals, usually by hematologists and cardiologists. Our laboratory has acted as an informal Swedish referral center for biopsy diagnosis of amyloidosis. The Swedish National Board of Health and Welfare has recently decided to create up to four National Highly Specialized Health Care Units for systemic amyloidosis, of which two should be able to perform advanced tissue-based diagnostic work (49).

A variety of diagnostic principles have been developed, each with advantages and drawbacks. ATTRwt amyloidosis offers particular difficulties. The deposits in peripheral tissue, such as in a subcutaneous fat pad, can be minute and very difficult to identify, and determining the amyloid type of such small particles is a challenge, well illustrated in our material. IHC solved the nature in many cases, but for the remaining patients in whom endomyocardial biopsy was unsuitable, the ATTR diagnosis had to rely on the typically sparse deposits in subcutaneous fat tissue combined with clinical methods, including imaging. This should also be true for patients with negative fat tissue biopsy but with a strong suspicion of ATTRwt amyloidosis.

The difficulties with ATTRwt amyloidosis have made a cardiac scintigraphy method attractive. This method is based on the affinity for skeletal markers to ATTR amyloid-containing hearts. In Europe, [^99m^Tc]Tc-labeled 3,3-disphos-phono-1,2-propanodicarboxylic acid (DPD) is most commonly used. The method is fairly specific for ATTR amyloidosis when AL disease has been excluded. However, the affinity of DPD is not due to the amyloid itself, as was originally believed but depends on irregularly distributed dense clouds of microcalcifications (50). Therefore, DPD scintigraphy is a surrogate method rather than a way to truly demonstrate amyloid. This fact is particularly important to know when trying to show therapeutic effects on the degree of heart involvement.

The field of systemic amyloidosis is developing rapidly, and treatment has become more individualized. It is probable that not only the biochemical type will have to be determined in the future but also subtypes, as is already the case with ATTRV30M amyloidosis. A reasonable subject might be AL amyloidosis, in which the amyloid protein originates from one out of around 50 possible immunoglobulin light chain genes. There are indications that certain gene products are associated with depositions in specific organs (51). Since AL proteins are cleaved and mainly consist of the variable region and a usually short part of the constant segment, fragmentation may also be important in amyloid fibril protein properties (52, 53).

There are other, even more, rare forms of systemic amyloidosis, some hereditary due to mis-sense mutations and some of wild-type nature. So far, we have not seen such examples in our recent material. However, the first AApoAIV case described was found in our material (54), and more unexpected cases will probably turn up.

In summary, immunological methods to determine the type of amyloidosis are safe and cheap but demand experience and specific antibodies that are not commercially available. For some materials, mass spectrometry is necessary. There are also cases with very little amyloid in the subcutaneous fat tissue for which other methods are of value, particularly for ATTRwt amyloidosis.
